# Influence of community disadvantage on cognitive performance in son-mother dyads in Duchenne muscular dystrophy

**DOI:** 10.1093/braincomms/fcag306

**Published:** 2026-08-20

**Authors:** Hakinya Karra, Varsha S Penumalee, Shreya Arun, Sanjana Javalkar, Genila Bibat, Matthew Ridder, Aaron J Kaat, Mathula Thangarajh

**Affiliations:** Department of Neurology, Virginia Commonwealth University, Richmond, VA 23298, USA; Department of Neurology, Virginia Commonwealth University, Richmond, VA 23298, USA; Department of Neurology, Virginia Commonwealth University, Richmond, VA 23298, USA; Department of Neurology, Virginia Commonwealth University, Richmond, VA 23298, USA; Center for Genetic Muscle Disorders, Kennedy Krieger Institute, and Johns Hopkins School of Medicine, Baltimore, MD 21205, USA; Department of Neurology, Virginia Commonwealth University, Richmond, VA 23298, USA; Department of Medical Social Sciences, Northwestern University Feinberg School of Medicine, Chicago, IL 60611, USA; Department of Neurology, Virginia Commonwealth University, Richmond, VA 23298, USA

**Keywords:** DMD; Duchenne muscular dystrophy, cognition, community disadvantage, social determinants of health

## Abstract

More than 80% of health outcomes are determined by social determinants of health. That social determinants of health affect physical health outcomes, as shown in previous studies of Duchenne muscular dystrophy, a progressive X-linked recessive genetic disease caused by variants in the *DMD* gene, in which males are affected whereas females are disease carriers. To date, however, whether community-level disadvantage affects brain health in Duchenne muscular dystrophy has not been investigated. We addressed this question by measuring cognitive performance of 65 males with Duchenne muscular dystrophy and their 60 biological mothers recruited from a national pool of study participants, hypothesizing that community disadvantage would negatively correlate with cognitive performance. The social vulnerability index—a geospatially determined scale—was used to estimate the degree of community disadvantage experienced by each son-mother dyad using their residential zip code. Total, crystallized, and fluid composite cognition scores were obtained from all study participants using an age-appropriate battery of cognition with the National Institutes of Health Toolbox Cognition Battery. The location of Duchenne muscular dystrophy variants from affected Duchenne muscular dystrophy males and the Duchenne muscular dystrophy carrier status of their biological mothers were collected as part of the study protocol. The mean age of the 65 males with Duchenne muscular dystrophy was 10.7 years [standard deviation 3.6], and their 60 biological mothers were 42.0 years [standard deviation 7.0]. Males with Duchenne muscular dystrophy scored lower than age-expected norms in fluid cognition (*n* = 57, mean = 82.7; standard deviation = 22.8), and within age-expected range in crystallized and total cognition (*n* = 56, mean = 105.4, standard deviation = 21.3; *n* = 55, mean = 93.5, standard deviation = 24.9, respectively). As a group, biological mothers scored in the normative range, except in one fluid cognition measure, namely the Flanker task of inhibitory control and attention. Biological mothers who were *DMD* carriers scored lower than non-carrier mothers in total composite and fluid cognition scores. Among the fluid cognition scores, *DMD* carriers scored lower in the Flanker task of inhibitory control and attention and cognitive flexibility. Son-mother dyads from communities with higher social vulnerability index scored lower on fluid cognition measures, namely the Flanker task of inhibitory control and attention (*r* = −0.20) and the dimensional card change sort test of cognitive flexibility (*r* = −0.16), respectively. Males with Duchenne muscular dystrophy and their biological mothers from disadvantaged communities demonstrate vulnerability in cognitive measures that are critical for attention regulation and cognitive flexibility. Our data highlighted the influence of the experiential social environment in Duchenne muscular dystrophy.

## Teaser

Karra *et al*. used a national geospatial index of resource scarcity and found that community disadvantage is negatively correlated with inhibitory control in sons with Duchenne muscular dystrophy and with cognitive flexibility in biological mothers. Community disadvantage negatively affects cognitive health in Duchenne muscular dystrophy.

## Graphical Abstract

**Graphical Abstract:**
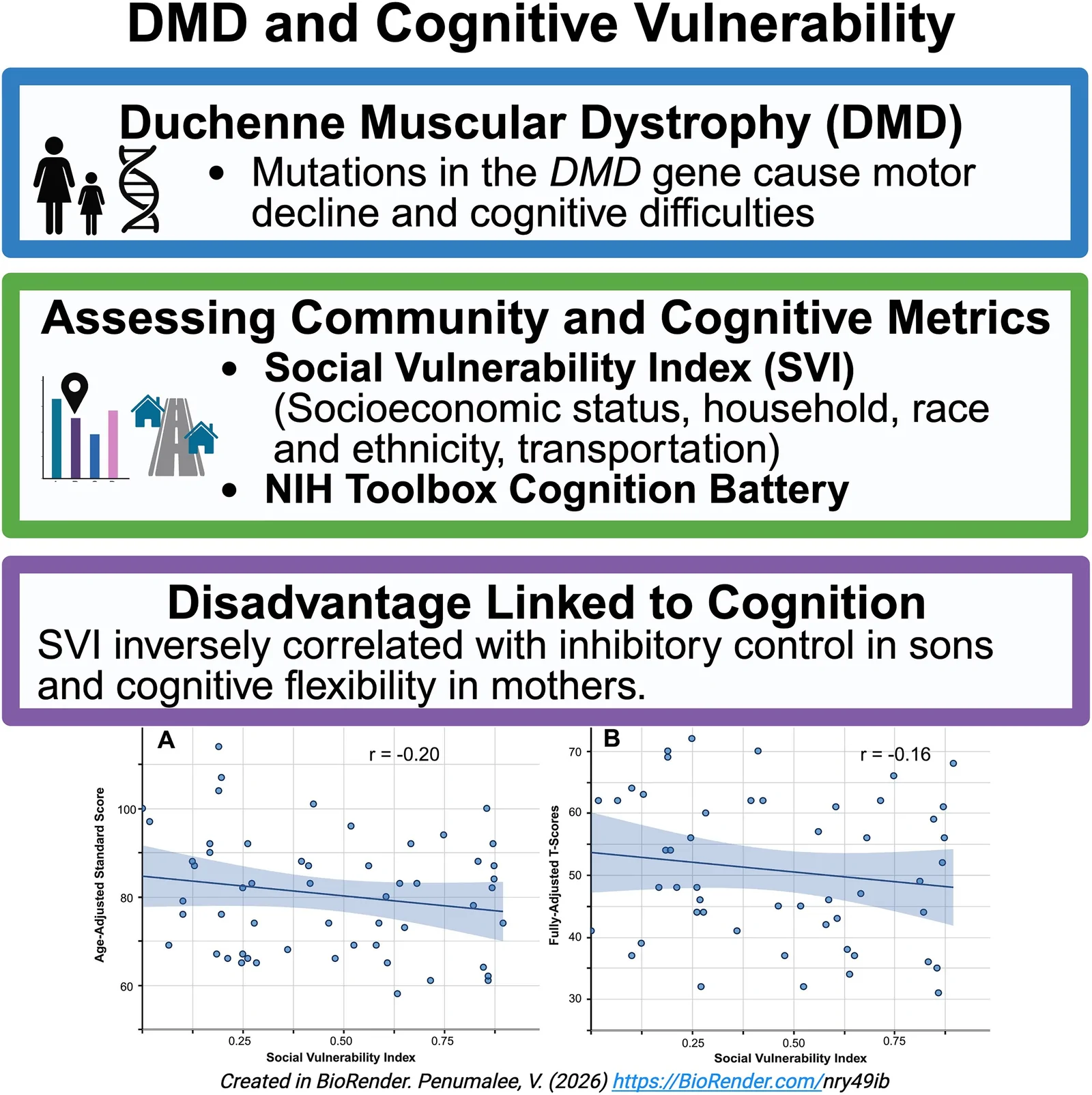

## Introduction

Childhood is a period of exploration, learning and growth. Epidemiological studies have clearly shown that the immediate environments of children critically shape their cognitive and socio-emotional development.^1,2^ Children growing in a state of deprivation—physical, cognitive and emotional—are at a higher risk of experiencing psychosocial stress, poor health outcomes and mental health diseases.^3,4^ Social determinants of health (SDOH) are ‘the conditions in the environments where people are born, live, learn, work, play, worship and age that affect a wide range of health, functioning and quality-of-life outcomes and risks’.^5^ Understanding the mechanisms by which SDOH affect child health is an active area of research. The availability of nationally normed indices of community disadvantage offers an opportunity to investigate experiential factors that can affect cognitive health. Furthermore, such knowledge can be used for community-level interventions and can inform public health policies.

Duchenne muscular dystrophy (DMD), affecting approximately one in 3500–5000 live male births worldwide, is the most common X-linked recessive genetic disorder that is caused by out-of-frame mutations in the *DMD* gene. The molecular biomarker dystrophin is lacking in affected individuals.^6-9^ DMD is a multisystem disease, with both physical and cognitive challenges,^10,11^ and individuals with distal *DMD* variants experience a higher cognitive burden.^12,13^ Prior work has shown that affected males with DMD residing in high-poverty neighbourhoods experience diagnostic delay, barriers to disease-modifying therapy and clinical trial participation.^14-16^ To date, however, whether community disadvantage imposes a burden on cognitive performance has not been investigated in DMD. This knowledge gap is important to address, given the crucial role of the social environment in shaping a child’s cognitive and socio-emotional development.

Two-thirds of biological mothers are *DMD* mutation carriers.^17^ Carrier mothers perform worse psychometrically in tests of inhibitory control/attention, compared to non-carrier mothers,^18,19^ but whether a disadvantaged social environment negatively affects their cognitive performance has not been previously investigated.

**Figure 1:**
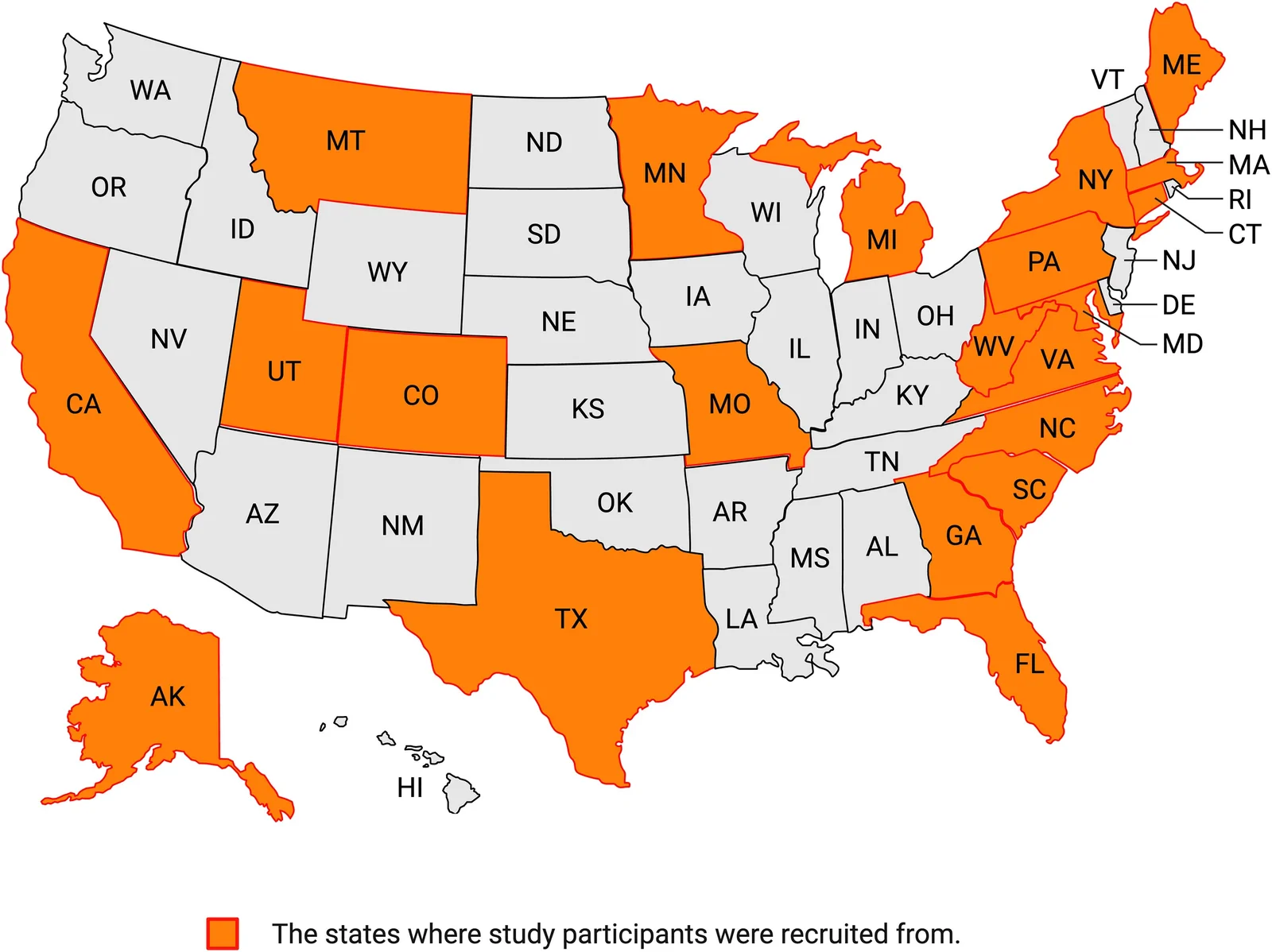
**Geographic distribution of participants by state of origin.** A map of the continental United States depicting the states from where study participants were residing at the time of study enrolment. The map was created using BioRender (*Penumalee, V. (2026)*  https://BioRender.com/wa9l00z).

Nationally normed indices that provide an inclusive estimate of both the home environment and the community can be used to measure social advantage/disadvantage. We applied the SDOH framework to investigate cognitive performance in individuals with DMD and their biological mothers, hypothesizing that dyads from disadvantaged communities may score lower on tests of cognitive performance. We tested our hypothesis in a prospective study cohort recruited from a national pool of participants (Fig. 1).

## Materials and methods

### Study design

This prospective study enrolled participants from 3 November 2017 to 15 November 2023 from three academic institutions. Each Institutional Review Board approved the study. Written informed consent was obtained from a parent/caregiver of all study participants; additional written assent was obtained from study participants older than 13 years of age.

### Participants

The study included 65 males and 60 females. Study subjects were 65 sons and their 60 biological mothers. Of the 65 dyads, five biological mothers each had two affected sons. All sons had a clinical diagnosis of DMD and/or a confirmed pathogenic *DMD* variant. Out-of-state study participants were fully reimbursed for study visit-related expenses. All sons received a nominal gift card for their participation in the study.

### *DMD* genotype in sons and carrier status in biological mothers

*DMD* variant information from sons and *DMD* carrier status from biological mothers were collected as part of study procedures. *DMD* variants were defined as proximal when located proximal to the 5ʹ *DMD* intron 44 and distal when they included nucleotides 3ʹ *DMD*, including intron 44, as previously described.^20^

### Community disadvantage

The social vulnerability index (SVI) is a nationally normed index of community disadvantage. It is calculated based on zip code tabulation areas (ZCTAs) from an aggregate of four main categories: (i) socioeconomic status (below poverty, unemployed, income and no high school diploma); (ii) household composition and disability (age 65 or older, age 17 or younger, older than age 5 with a disability and single-parent households); (iii) minority status and language (minority, speaks English ‘less than well’); and (iv) housing and transportation (multiunit structures, mobile homes, crowding, no vehicle and group quarters).^21^

Each study dyad was assigned a score from zero to one on the SVI, with zero representing the least vulnerable and one being highly vulnerable. Furthermore, dyads were assigned to one of four quartiles based on their SVI; the first to fourth quartiles represent ‘low vulnerability’, ‘low-medium vulnerability’, ‘medium-high vulnerability’ and ‘high vulnerability’, respectively.^22-24^ Zip codes were available for 58 of 65 dyads (89%) and were collected as part of study records. Zip codes were then converted to ZCTAs to obtain individual SVI scores.

### Tests of cognitive assessment

Cognitive performance was assessed using the National Institutes of Health Toolbox for the Assessment of Neurological and Behavioral Function Cognition Battery (NIHTB-CB), a population-normed neurobehavioural measure.^25,26^ The NIHTB-CB provides three composite scores: crystallized cognition, fluid cognition and total cognition scores. Crystallized cognition scores are a normalized average of two tests: vocabulary (picture vocabulary) and reading (oral reading recognition). Fluid cognition scores are a normalized average of scores from five tests: task switching [dimensional change card sort (DCCS)], episodic memory [picture sequence memory (PSM)], working memory [list sorting working memory (LSWM)], processing speed [pattern comparison (PCPS)] and inhibitory control (flanker inhibitory control and attention). Total cognition scores are the normalized averages of fluid and crystallized cognition scores. Age-corrected standard scores for sons [mean = 100, standard deviation (SD) = 15] and demographically adjusted T-score norms for the biological mothers (mean = 50, SD = 10) were used.

### Statistical analysis

All analyses were conducted in the R statistical language. For comparisons by *DMD* variant location (proximal versus distal), the sons’ age-adjusted NIHTB-CB scores were compared using the nonparametric Mann–Whitney U-test with a dichotomous indicator for *DMD* variant location. Likewise, for maternal comparisons, the mothers’ demographically adjusted NIHTB-CB scores were compared using the Mann–Whitney U-test for carrier status. For comparisons with the SVI score, simple linear regression was utilized with SVI as the single predictor for performance on the NIHTB-CB. As these analyses were largely exploratory insofar as they had not been considered previously, no type-I error corrections were utilized. R statistical software used for data analysis is available in the Supplementary materials.

## Results

### Demographic characteristics

The demographic characteristics of the dyads are described in Table 1. A total of 65 sons and 60 biological mothers were evaluated, and Fig. 1 illustrates the states from the continental USA from where study participants were recruited. The mean age of sons was 10.7 years (SD = 3.6; range: 4–22 years), and the mean age of biological mothers was 42 years (SD = 7.0; range: 26–59 years). Racial data were available for all sons, and ethnicity data were available for 63 of 65 sons (97%). Study participants self-reported race as White (54/65), Asian (5/65), African American (3/65), American Indian (1/65) or biracial (2/65) and ethnicity as non-Hispanic (53/63) and Hispanic (10/63).

**Table 1:** Clinical and demographic characteristics of study dyads

Sons (*n* = 65)		Biological mothers (*n* = 60)	
Mean age in years (range)	10.7 (4–22)	Mean age in years (range)	42.0 (26–59)
Location of *DMD* mutation		DMD carrier status	
Proximal	30	Carrier	30
Distal	30	Non-carrier	19
Unknown	5	Unknown	11
Race		Race	
White	54	White	49
Asian	5	Asian	5
African American	3	African American	3
American Indian	1	American Indian	1
Biracial	2	Biracial	2
Ethnicity		Ethnicity	
Not Hispanic	54	Not Hispanic	50
Hispanic	10	Hispanic	7
Unknown	1	Unknown	3
Type of *DMD* Mutation		Household income	
Deletion	34	<50K	12
Nonsense	20	50–100K	17
Point mutation	4	100–150K	14
Duplication	3	>150K	13
Unknown	4	Unknown	4
Co-occurring conditions (*n* = 61)		Employment status	
Attention-deficit hyperactivity disorder	6	Employed	33
Anxiety	2	Not employed	18
Autism spectrum disorder	1	Unknown	9
Developmental dyscoordination syndrome	1		
No other medical condition	53		
Oral corticosteroid use (*n* = 61)			
Deflazacort	36		
Prednisone	10		
Vamorolone	2		
Unspecified corticosteroid	2		
No current corticosteroid use	11		

*DMD* variant information was available in 62 of 65 sons (95%). *DMD* proximal and distal variants were equally distributed, 31 of 62 sons (50%) in each. The type of *DMD* variant was available in 61 of 65 sons (94%). The *DMD* variants included deletion mutations (34/61), nonsense mutations (20/61), point mutations (4/61) and duplications (3/61). The standard-of-care for DMD is oral corticosteroids, which was reported in 61 of 65 sons (94%), of whom 36 reported taking deflazacort, 10 were on prednisone and 2 were on the dissociative steroid, vamorolone. Two study participants did not report the name of the corticosteroid prescribed to them. Eleven males with DMD were corticosteroid naive. Concurrent diagnoses were reported in 61 of 65 sons (94%), with six reporting attention-deficit hyperactivity disorder, two with anxiety disorder, one with autism spectrum disorder and one with developmental coordination disorder. *DMD* carrier status was available for 49 biological mothers. Thirty of the 49 (61%) biological mothers were carriers of the *DMD* variant.

### Cognitive performance of dyads on the NIHTB-CB

**Figure 2:**
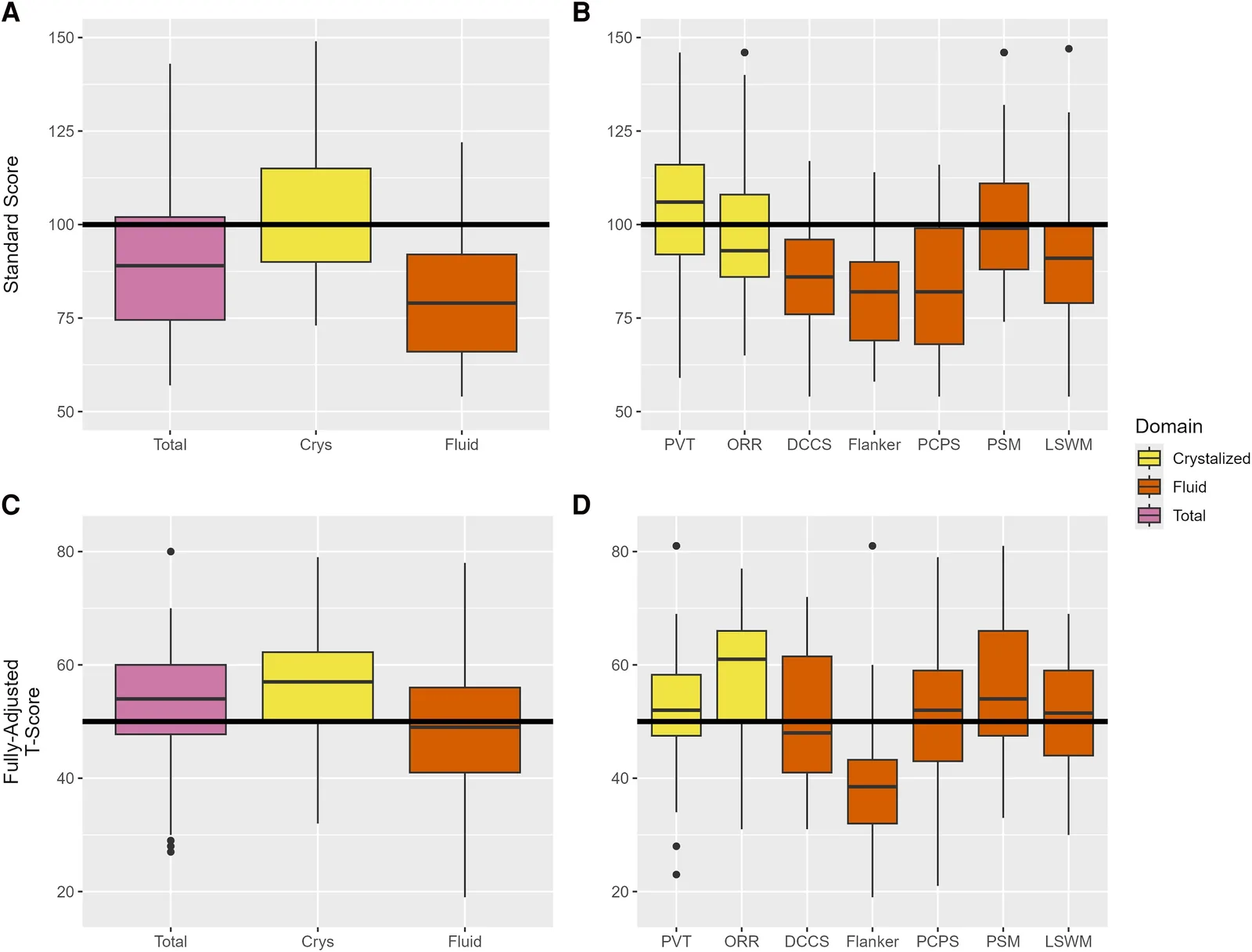
**Graphical representation of performance of son-mother dyads with *DMD* variants on cognitive domains as assessed by the National Institutes of Health Toolbox—Cognition Battery (NHTB-CB).** (**A**) Sixty-five sons’ age-corrected NIHTB-CB total cognition, crystalized cognition, and fluid cognition scores. (**B**) Sons’ age-corrected NIHTB-CB crystalized and fluid cognition subtest scores. (**C**) Sixty mothers’ fully corrected NIHTB-CB total cognition, crystalized cognition and fluid cognition T-scores. (**D**) Mothers’ fully corrected NIHTB-CB total cognition, crystalized cognition and fluid cognition subtest T-scores. The sons’ scores are age-adjusted standard scores, with a mean of 100 and a sample SD of 15. The mothers’ score was calculated using a demographically adjusted T-score using age, sex, race/ethnicity and years of education as variables, with a mean of 50 and a sample SD of 10. Each data point represents individual performance on the NIHTB-CB battery. PVT, ORR, DCCS), Flanker Task of Inhibitory Control and Attention (Flanker), PCPS, PSM and LSWM. The sons’ total cognition score (*n* = 55, mean = 90.5, SD = 20.1), crystallized cognition scores (*n* = 56, mean = 103.2, SD = 18.9) and fluid cognition score (*n* = 57, mean = 80.1, SD = 17.5) are shown (Fig. 2A and B). The biological mothers (*n* = 60) performed below expectations on the demographically adjusted T-scores in Flanker (Fig. 2C and D). The line within the box depicts the median, and the box represents inter-quarter range (25th and 75th) of age-correct standard scores. The outlier values are indicated as dark dots.

The sons’ total cognition and crystallized cognition scores was at par with age-expected norms (*n* = 55, mean = 90.5, SD = 20.1; *n* = 56, mean = 103.2, SD = 18.9, respectively) (Fig. 2A). On fluid cognition, the sons scored lower than age-expected norms (*n* = 57, mean = 80.1, SD = 17.5) (Fig. 2A). Within the fluid cognition scores, the lowest performance was detected on Flanker and PCPS (Fig. 2B). Within the crystallized cognition scores, performance was below age-expected norms in oral reading recognition (Fig. 2B).

The overall performance of biological mothers on the NIHTB-CB was within the expected range (Fig. 2C), except for Flanker, where they scored below expectations on the demographically adjusted T-scores (Fig. 2D).

### Cognitive performance of sons as a function of DMD variant location

**Figure 3:**
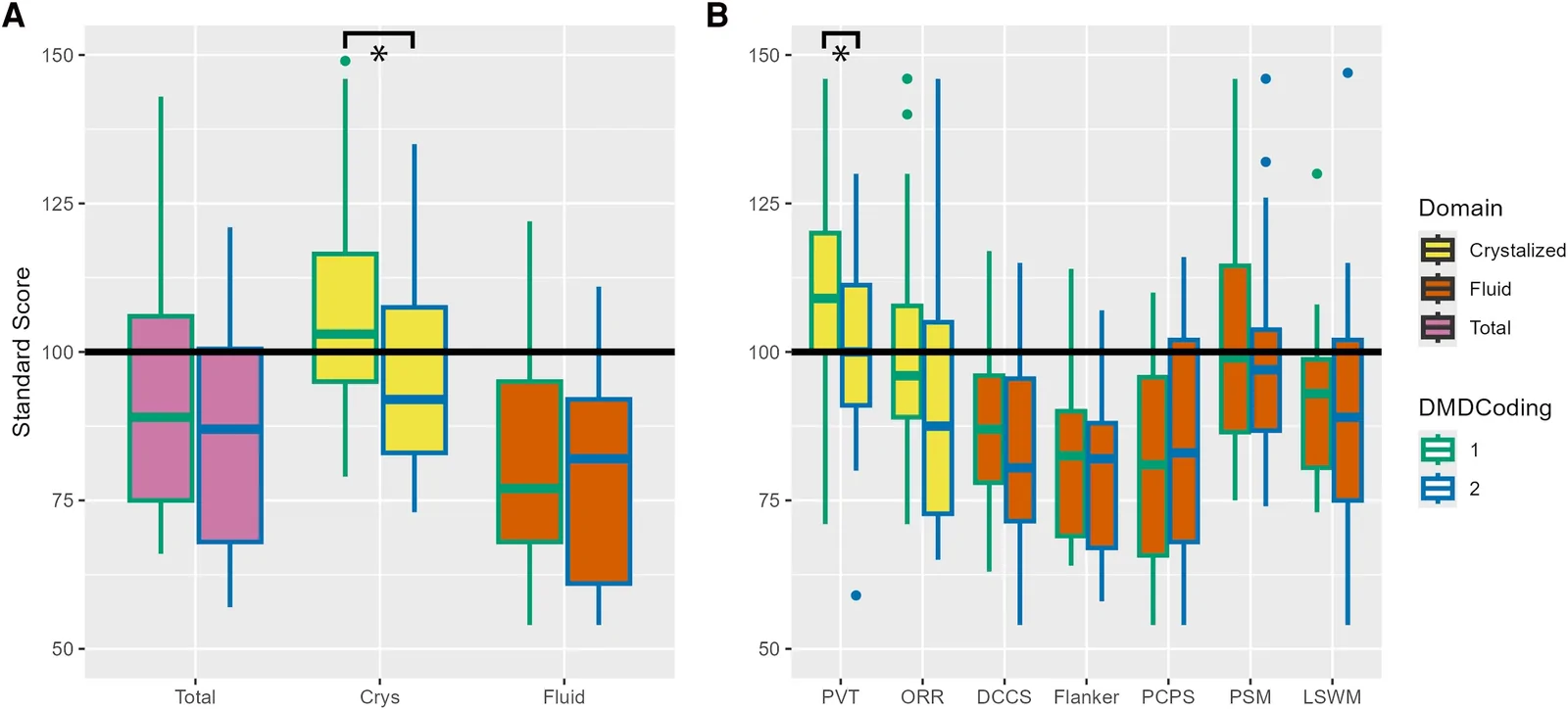
**Performance of sons (*n* = 61) on cognitive domains as assessed by the NHTB-CB as a function of *DMD* variant location.** (**A**) Age-corrected NIHTB-CB total cognition, crystalized cognition, and fluid cognition scores of sons with proximal (1) versus distal (2) *DMD* variants. (**B**) Age-corrected NIHTB-CB crystalized and fluid cognition subtest scores of sons with proximal (1) *versus* distal (2) *DMD* variants. The box plot shows the median and inter-quarter range. Statistical comparisons between groups are provided in Table 2. PVT, ORR, DCCS), Flanker Task of Inhibitory Control and Attention (Flanker), PCPS, PSM and LSWM. For comparison of *DMD* variant location (proximal versus distal), the sons’ age-adjusted NIHTB-CB scores were compared using the nonparametric Mann–Whitney U-test with a dichotomous indicator for *DMD* variant location. The line within the box depicts the median, and the box represents inter-quarter range (25th and 75th) of age-correct standard scores. The outlier values are indicated as dark dots. Statistically significant difference in performance was detected on the crystalized cognition score (*) (Fig. 3A), driven by performance difference in the picture vocabulary domain.

The sons’ performance on the NIHTB-CB was investigated as a function of *DMD* variant location. Sons with proximal *DMD* variants, compared to sons with distal *DMD* variants, scored higher on total cognition and crystallized cognition, with statistically significant differences in crystallized composite (*P* = 0.03), and on picture vocabulary (*P* = 0.02; Table 2 and Fig. 3A and B). Both groups performed similarly on fluid cognition (Fig. 3B).

**Table 2:** Differences in NIHTB-CB age-adjusted scores^a^ by location of DMD variant

NIHTB-CB test domain	Number of study participants with proximal *DMD* mutations	Median value with range for participants with proximal *DMD* mutations	Number of study participants with distal *DMD* mutations	Median value with range for participants with distal *DMD* mutations	*P*-value	Location difference	95% Confidence interval
Picture vocabulary	31	109 [20; 71, 146]	30	100 [20.2; 59–130]	0.02*	10.0	1.0–18.0
Oral reading	30	96 [19; 71–146]	23	89 [34; 65–151]	0.09	11.0	−1.0 to 21.0
Crystallized composite	30	103 [21; 79–149]	23	92 [24; 73–135]	0.03*	12.0	1.0–20.0
Flanker	30	82.5 [21; 64–114]	29	82 [21; 58–107]	0.61	2.0	−7.0 to 8.0
LSWM	30	93 [18; 73–130]	25	89 [27; 54–147]	0.38	3.0	−5.0 to 13.0
DCCS	31	87 [18; 63–117]	30	80 [24; 54–115]	0.16	5.0	−3.0 to 13.0
PCPS	30	81 [30; 54–110]	25	83 [34; 54–116]	0.38	−4.0	−15.0 to 6.0
PSM	31	99 [28; 75–146]	30	97 [17; 74–146]	0.43	3.6	−5.0 to 13.0
Fluid composite	29	77 [27; 54–122]	25	82 [31; 54–111]	0.85	1.0	−10.0 to 11.0
Total composite	29	89 [31; 66–143]	23	87 [32; 57–121]	0.32	7.0	−7.0 to 17.0

^a^Score differences utilize age-adjusted standard scores.

^*^ *P* < 0.05.

### Cognitive performance of mothers as a function of *DMD* carrier Status

**Figure 4:**
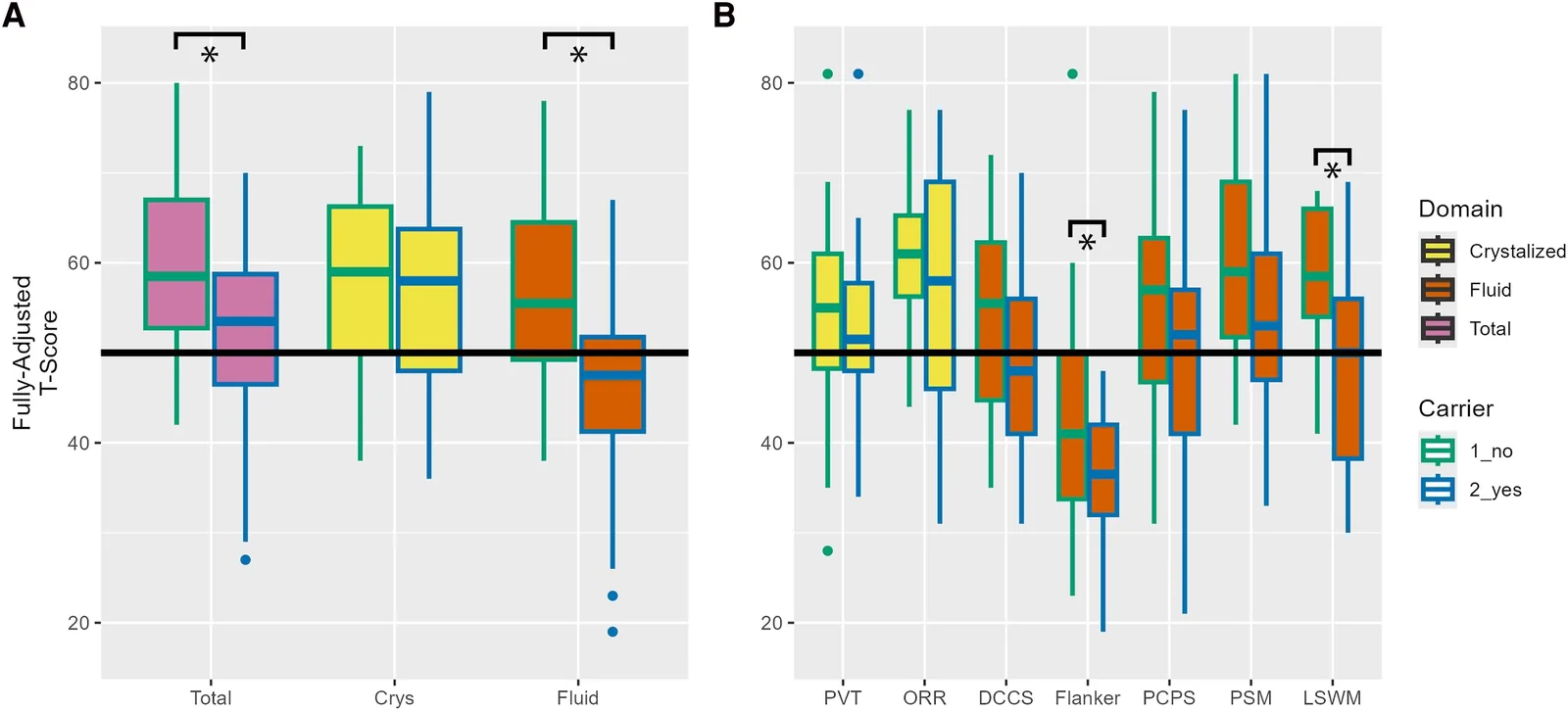
**Performance of mothers (*n* = 54) on cognitive domains as assessed by the NHTB-CB as a function of known *DMD* carrier status.** (**A**) Fully corrected NIHTB-CB total cognition, crystalized cognition, and fluid cognition T-scores of non-carrier (1) versus carrier (2) mothers. (**B**) Fully corrected NIHTB-CB crystalized and fluid cognition subtest T-scores of non-carrier (1) versus carrier (2) mothers. The box plot shows the median and inter-quarter range. Statistical comparisons between groups are provided in Table 3. PVT, ORR, DCCS, Flanker Task of Inhibitory Control and Attention (Flanker), PCPS, PSM and LSWM. The mothers’ demographically adjusted NIHTB-CB scores were compared using the Mann–Whitney U-test with a dichotomous indicator for the carrier status. The line within the box depicts the median, and the box represents inter-quarter range (25th and 75th) of age-correct standard scores. The outlier values are indicated as dark dots. Statistically significant performance was detected on Total and Fluid cognition scores (*). The differences in Fluid cognition score were in the domains of LSWM and Flanker task of inhibitory control and attention Flanker (*).

The mothers’ performance on the NIHTB-CB was investigated as a function of *DMD* carrier status. Carrier mothers scored lower in total composite (*P* = 0.03) and fluid cognition scores (*P* = 0.003) compared to non-carrier mothers (Table 3 and Fig. 4A and B). In all the individual tests of fluid cognition, carrier mothers scored lower than for non-carriers with statistically significant difference in Flanker Task of Inhibitory Control and Attention (0.04) and LSWM (*P* = 0.009).

**Table 3:** Differences in NIHTB-CB performance^a^ for mothers by carrier status

NIHTB-CB test domain	Number of carrier mothers	Median value with range for carrier mothers	Number of non-carrier mothers	Median value with range for non-carrier mothers	*P*-value	Difference	95% confidence interval
Picture vocabulary	30	52.5 [10.5; 34–81]	19	55 [13.5; 28–81]	0.56	2.0	−5.0 to 7.0
Oral reading	29	61 [21; 31–77]	19	61 [10; 44–77]	0.86	0.0	−6.0 to 8.0
Crystallized composite	30	58.5 [14.25; 36–79]	19	59 [16.5; 38–73]	0.54	2.0	−5.0 to 8.0
Flanker	30	37.5 [10; 19–48]	19	41 [14; 23–81]	0.04*	5.0	1.1e-5–11.0
LSWM	30	50 [17; 30–69]	19	58 [11.5; 41–68]	0.009*	8.0	3.0–15.0
DCCS	29	48 [15; 31–70]	19	57 [18; 35–72]	0.12	6.0	−1.0 to 13.0
PCPS	29	52 [17; 21–77]	19	56 [20; 31–79]	0.25	5.0	−3.0 to 14.0
PSM	29	53 [16; 33–81]	19	60 [16.5; 42–81]	0.07	7.0	−1.3e-5 to 15.0
Fluid composite	30	47.5 [10.5; 19–67]	19	56 [16.5; 38–78]	0.005*	10.0	3.0–17.0
Total composite	30	53.5 [12.5; 27–70]	19	61 [14.5; 42–80]	0.021*	7.0	1.0–14.0

^a^Score differences utilize fully-adjusted T-scores.

^*^ *P* < 0.05.

### Cognitive performance of dyads and social environmental metrics

**Figure 5:**
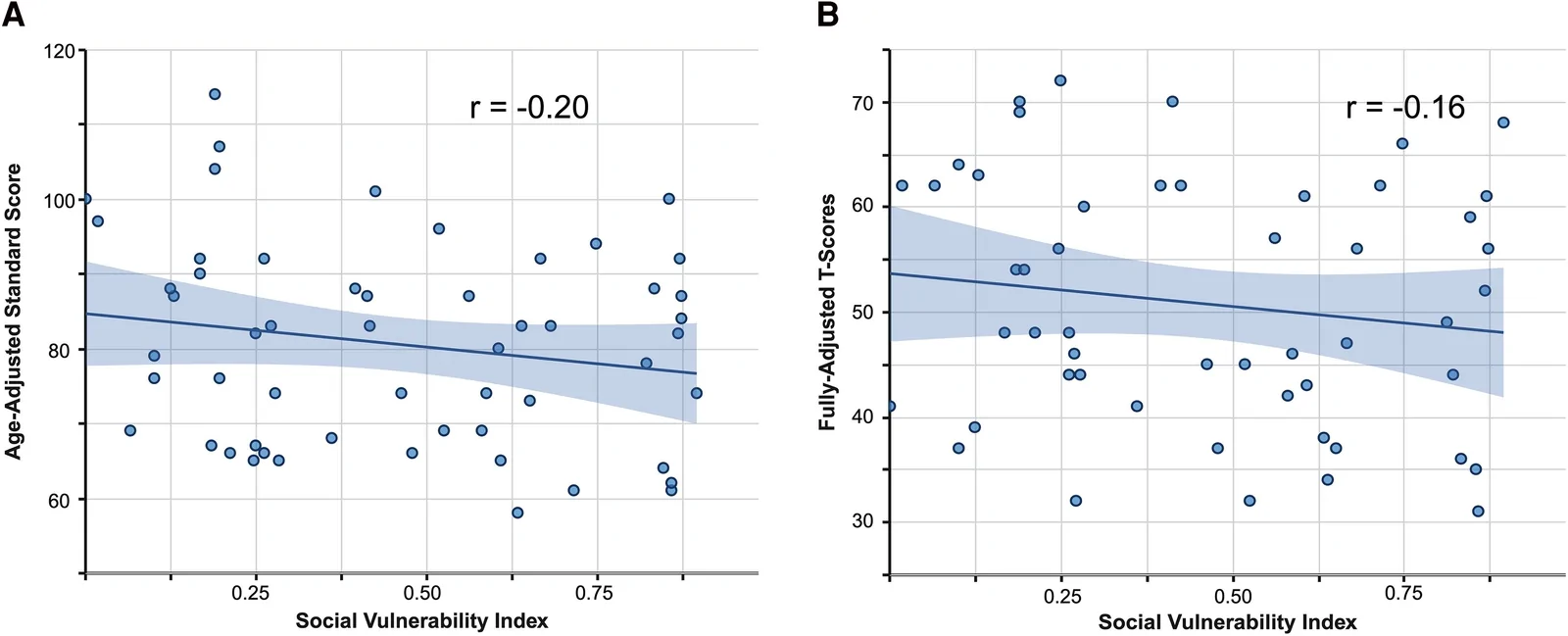
**Association between community disadvantage and cognitive performance in son-mother dyads.** (**A**) Bivariate linear regression (with standard error shaded in grey) between SVI and age-corrected **NHTB-CB** Flanker Task of Inhibitory Control/Attention scores in sons with DMD. (**B**) Bivariate linear regression between SVI and fully corrected NIHTB-CB DCCS test of cognitive flexibility T-scores in biological mothers. The number of son-mother dyads analysed was 58, and each data point represents a son-mother dyad. The figure was created using BioRender (*Penumalee, V. (2026) https://BioRender.com/6bnfg8i).*

SVI was available from 58 of the 65 dyads (89%). Seventeen dyads (29%) were from communities of low vulnerability (SVI 1), 15 (26%) from low to medium vulnerability (SVI 2), 13 (22%) were from medium to high vulnerability (SVI 3) and 13 (22%) (SVI 4) were from high vulnerability. Dyads demonstrated a negative correlation between SVI and cognitive performance. With increasing SVI, sons scored lower on Flanker (*r* = −0.20; Fig. 5A), whereas mothers scored lower on DCCS (*r* = −0.16; Fig. 5B).

## Discussion

Cognitive challenges are increasingly recognized as a major determinant of quality of life in individuals with DMD,^27^ but whether SDOH affect their cognitive performance has not yet been investigated. Because of the positive correlation between child and mother cognitive performance, this cross-sectional study aimed to investigate the relationship between community disadvantage, as indexed by the SVI, and cognitive performance in 65 sons with DMD and their 60 biological mothers. We found that dyads from socially disadvantaged communities demonstrated lower cognitive performance. These findings align with prior epidemiological and psychological studies that have demonstrated the link between economic prosperity and better educational^28^ and health outcomes.^29^

Inhibition and cognitive flexibility are two critical components of executive function skills, which are essential for navigating daily life. Inhibition involves suppressing impulsive responses and resisting distractions to complete goal-directed behaviour. Cognitive flexibility, on the other hand, is the ability to adapt to changing environments, switch between tasks and adjust behaviour based on new information. These functions are interconnected and crucial for managing complex tasks, but disruptions in these processes are common in various diseases, including DMD.^30^ In individuals with DMD, executive function challenges may be exacerbated by both genetic factors—such as variations in *DMD* genotype—and environmental stressors. Previous studies have shown that individuals with distal *DMD* mutations may exhibit greater deficits in digit span, visuospatial function, visual memory and syntactic processing.^12,31^ Carrier mothers perform worse on assessments of attention, working memory, immediate verbal memory and visuospatial skills than non-carrier mothers.^18,19^ Mothers caring for children with DMD in a resource-limited setting may also face additional cognitive and emotional stress, which could further impair their ability to support their child’s cognitive development.^32,33^

There is evidence that brain networks subserving executive functions are shaped by socioeconomic factors. Changes in brain connectivity involving the prefrontal cortex^34^ and the anterior cingulate cortex,^35^ responsible for cognitive control and socioemotional responses, respectively, have been shown to be associated with social disadvantage. For example, Kishiyama *et al.*^36^ found that children from lower socio-economic status (SES) demonstrated reduced prefrontal cortical activation during concurrent performance of two relatively simple tasks (i.e. task of interference), compared to their peers from higher SES. Similarly, Huijsmans *et al.*^37^ highlighted that a scarcity mindset can alter brain connectivity, threatening goal-directed behaviour. Behavioural research into cognitive scarcity has also shown that individuals facing resource constraints may experience cognitive overload, resulting in diminished cognitive resources available for tasks requiring executive control.^38,39^

One socioeconomic factor known to predict cognitive outcomes in children is maternal education. It has been established that higher maternal education levels correlate with better developmental outcomes for offspring.^32,40^ Poor household composition has also been previously shown to affect cognitive development in children during developmentally sensitive epochs.^41^ This is consistent with previous findings, such as those by Fowler *et al.,*^42^ which suggested that unstable home environments may affect cognitive development in children between the ages of four and 14. Similarly, Baker *et al.*^43^ found that housing instability is associated with poorer psychosocial and physical health outcomes, particularly among children with lower baseline physical health. These findings emphasize the importance of stable and supportive household conditions for cognitive health, suggesting that interventions aimed at improving the home environment may mitigate the effects of community disadvantage on cognitive performance, including in DMD.

Extant literature supports the notion that socioeconomic factors significantly affect outcomes in DMD, including prevalence, diagnosis, access to medical care, uptake of disease-modifying therapies and survival rates.^15,44,45^ Bushby *et al.*^46^ used the Townsend scale, a measure of social deprivation, to link DMD prevalence with levels of social deprivation. They found that areas of Northern England with greater levels of social deprivation had a higher prevalence of DMD. Although the underlying scientific cause of this association has not been explored, this finding highlights the role of environmental factors in disease prevalence and incidence.

To our knowledge, this is the first study to demonstrate an association between community disadvantage and cognitive performance in families affected by DMD. Our findings underscore the importance of experiential factors and understanding contextual barriers faced by families affected by DMD. These barriers, influenced by SDOH, have the potential to affect clinical care as well as the quality of life of affected individuals. Neuromuscular care teams can incorporate routine screening for SDOH to better identify and address SDOH in clinical practice. By integrating a broader approach that includes the social, economic and environmental challenges families face, healthcare providers would be able to tailor individualized medical care. Advocating for vital resources for families, including emotional and financial support, access to community-based interventions, can be pivotal in mitigating social disadvantages.

## Limitations

We acknowledge the limitations of our study. First, while our cohort size is large for a rare disease such as DMD (65 dyads), epidemiologically, this is a small sample size, which may limit the generalizability of our study findings. Second, our cohort had a greater number of White participants (*n* = 53) compared to other racial demographics. Third, our study was a cross-sectional analysis with a focus on cognitive performance, hindering the ability to investigate how SDOH affect motor function in DMD. Fourth, we did not correct for potential type I errors in our statistical analysis. Future longitudinal well-powered studies addressing these limitations will better inform how SDOH affect both motor and cognitive health in DMD.

## Conclusion

This study provides scientific evidence that community disadvantage is associated with lower inhibitory control in sons with DMD and with cognitive flexibility in their biological mothers. These findings suggest that socioeconomic factors influence cognitive performance in DMD, consistent with evidence on the effects of social disadvantage on cognition. The results highlight the need for healthcare providers to consider SDOH in clinical care, emphasizing the importance of addressing environmental stressors to support cognitive and emotional well-being. Our study underscores the value of integrating SDOH assessments into clinical care, advocating for public health policy to reduce community disadvantage to improve the quality of life for families affected by DMD and promoting better health outcomes.

## Supplementary Material

fcag306_Supplementary_Data

## Data Availability

De-identified data will be shared upon reasonable request after due agreement. The statistical codes used for data analysis are available in the Supplementary material.

## References

[1] Berry D, Blair C, Willoughby M, et al Household chaos and children’s cognitive and socio-emotional development in early childhood: Does childcare play a buffering role? Early Child Res Q. 2016;34:115–127. 29720785 10.1016/j.ecresq.2015.09.003 PMC5926246

[2] Ferguson KT, Cassells RC, MacAllister JW, Evans GW. The physical environment and child development: An international review. Int J Psychol J Int Psychol. 2013;48(4):437–468. 10.1080/00207594.2013.804190 PMC4489931 23808797

[3] Nelson CA, Scott RD, Bhutta ZA, Harris NB, Danese A, Samara M. Adversity in childhood is linked to mental and physical health throughout life. BMJ. 2020;371:m3048. 33115717 10.1136/bmj.m3048 PMC7592151

[4] Webster EM . The impact of adverse childhood experiences on health and development in young children. Glob Pediatr Health. 2022;9:2333794X221078708. 10.1177/2333794X221078708 PMC8882933 35237713

[5] Office of Disease Prevention and Health Promotion . Social determinants of health. https://www.cdc.gov/about/priorities/why-is-addressing-sdoh-important.html.

[6] Passamano L, Taglia A, Palladino A, et al Improvement of survival in Duchenne muscular dystrophy: Retrospective analysis of 835 patients. Acta Myol. 2012;31(2):121–125. 23097603 PMC3476854

[7] Mah JK . An overview of recent therapeutics advances for Duchenne muscular dystrophy. Methods Mol Biol. 2018;1687:3–17. 29067652 10.1007/978-1-4939-7374-3_1

[8] Mah JK . Current and emerging treatment strategies for Duchenne muscular dystrophy. Neuropsychiatr Dis Treat. 2016;12:1795–1807. 27524897 10.2147/NDT.S93873 PMC4966503

[9] Kariyawasam D, D’Silva A, Mowat D, et al Incidence of Duchenne muscular dystrophy in the modern era; an Australian study. Eur J Hum Genet. 2022;30(12):1398–1404. 35754057 10.1038/s41431-022-01138-2 PMC9712523

[10] Fee RJ, Montes J, Hinton VJ. Executive functioning in the dystrophinopathies and the relation to underlying mutation position. J Int Neuropsychol Soc. 2019;25(2):146–155. 30511603 10.1017/S1355617718000942

[11] Anwar S, He M, Lim KRQ, Maruyama R, Yokota T. A genotype-phenotype correlation study of exon skip-equivalent in-frame deletions and exon skip-amenable out-of-frame deletions across the DMD gene to simulate the effects of exon-skipping therapies: A meta-analysis. J Pers Med. 2021;11(1):46. 33466756 10.3390/jpm11010046 PMC7830903

[12] D’Angelo MG, Lorusso ML, Civati F, et al Neurocognitive profiles in Duchenne muscular dystrophy and gene mutation site. Pediatr Neurol. 2011;45(5):292–299. 22000308 10.1016/j.pediatrneurol.2011.08.003 PMC3200430

[13] Tyagi R, Aggarwal P, Mohanty M, Dutt V, Anand A. Computational cognitive modeling and validation of Dp140 induced alteration of working memory in Duchenne muscular dystrophy. Sci Rep. 2020;10(1):11989. 32686699 10.1038/s41598-020-68381-9 PMC7371893

[14] Mathews KD, Conway KM, Gedlinske AM, et al Characteristics of clinical trial participants with Duchenne muscular dystrophy: Data from the muscular dystrophy surveillance, tracking, and research network (MD STARnet). Child Basel Switz. 2021;8(10):835. 10.3390/children8100835 PMC8534386 34682100

[15] Mann JR, Zhang Y, McDermott S, et al Racial and ethnic differences in timing of diagnosis and clinical services received in Duchenne muscular dystrophy. Neuroepidemiology. 2023;57(2):90–99. 36623491 10.1159/000528962 PMC10273877

[16] Jagers S . Duchenne muscular dystrophy: age, disease stage, and patient-reported quality of life. 2023. Walden Dissertations and Doctoral Studies. 15202. https://scholarworks.waldenu.edu/dissertations/15202

[17] Hostinar CE, Stellern SA, Schaefer C, Carlson SM, Gunnar MR. Associations between early life adversity and executive function in children adopted internationally from orphanages. Proc Natl Acad Sci U S A. 2012;109(Suppl 2):17208–17212. 23047689 10.1073/pnas.1121246109 PMC3477377

[18] Thangarajh M, Kaat AJ, Bibat G, et al The NIH Toolbox for cognitive surveillance in Duchenne muscular dystrophy. Ann Clin Transl Neurol. 2019;6(9):1696–1706. 31472009 10.1002/acn3.50867 PMC6764624

[19] Demirci H, Durmus H, Toksoy G, Uslu A, Parman Y, Hanagasi H. Cognition of the mothers of patients with Duchenne muscular dystrophy. Muscle Nerve. 2020;62(6):710–716. 32893363 10.1002/mus.27057

[20] Thangarajh M, Bello L, Gordish-Dressman H; CINRG-DNHS Investigators. Longitudinal motor function in proximal versus distal DMD pathogenic variants. Muscle Nerve. 2021;64(4):467–473. 34255858 10.1002/mus.27371 PMC8780240

[21] Flanagan BE, Hallisey EJ, Adams E, Lavery A. Measuring community vulnerability to natural and anthropogenic hazards: The centers for disease control and prevention’s social vulnerability index. J Environ Health. 2018;80(10):34–36. PMC7179070 32327766

[22] Hyer JM, Tsilimigras DI, Diaz A, et al High social vulnerability and “textbook outcomes” after cancer operation. J Am Coll Surg. 2021;232(4):351–359. 33508426 10.1016/j.jamcollsurg.2020.11.024

[23] Azap RA, Paredes AZ, Diaz A, Hyer JM, Pawlik TM. The association of neighborhood social vulnerability with surgical textbook outcomes among patients undergoing hepatopancreatic surgery. Surgery. 2020;168(5):868–875. 32800602 10.1016/j.surg.2020.06.032

[24] Abla H, Collins RA, Dhanasekara CS, Shrestha K, Dissanaike S. Using the social vulnerability index to analyze statewide health disparities in cholecystectomy. J Surg Res. 2024;296:135–141. 38277949 10.1016/j.jss.2023.12.031

[25] Weintraub S, Bauer PJ, Zelazo PD, et al I. NIH toolbox cognition battery (CB): Introduction and pediatric data. Monogr Soc Res Child Dev. 2013;78(4):1–15. 10.1111/mono.12031 PMC3954750 23952199

[26] Weintraub S, Dikmen SS, Heaton RK, et al Cognition assessment using the NIH Toolbox. Neurology. 2013;80(11 Suppl 3):S54–S64. 23479546 10.1212/WNL.0b013e3182872ded PMC3662346

[27] Ueda Y . Cognitive function and quality of life of muscular dystrophy [Internet]. In: Muscular dystrophies. IntechOpen; 2019. 10.5772/intechopen.86222

[28] Broer M, Bai Y, Fonseca F. A review of the literature on socioeconomic status and educational achievement. In: Broer M, Bai Y, Fonseca F, eds. Socioeconomic inequality and educational outcomes: Evidence from twenty years of TIMSS. Springer International Publishing; 2019:7–17.

[29] Feinstein JS . The relationship between socioeconomic Status and health: A review of the literature. Milbank Q. 1993;71(2):279–322. 8510603

[30] Logue SF, Gould TJ. The neural and genetic basis of executive function: Attention, cognitive flexibility, and response inhibition. Pharmacol Biochem Behav. 2014;123:45–54. 23978501 10.1016/j.pbb.2013.08.007 PMC3933483

[31] Thangarajh M, Elfring GL, Trifillis P, McIntosh J, Peltz SW. The relationship between deficit in digit span and genotype in nonsense mutation Duchenne muscular dystrophy. Neurology. 2018;91(13):e1215–e1219. 30135256 10.1212/WNL.0000000000006245 PMC6161548

[32] Jackson M, Kiernan K, McLanahan S. Maternal education, changing family circumstances, and children’s skill development in the United States and UK. Ann Am Acad Pol Soc Sci. 2017;674(1):59–84. 29563643 10.1177/0002716217729471 PMC5857959

[33] Troller-Renfree SV, Costanzo MA, Duncan GJ, et al The impact of a poverty reduction intervention on infant brain activity. Proc Natl Acad Sci U S A. 2022;119(5):e2115649119. 35074878 10.1073/pnas.2115649119 PMC8812534

[34] Lawson GM, Duda JT, Avants BB, Wu J, Farah MJ. Associations between children’s socioeconomic status and prefrontal cortical thickness. Dev Sci. 2013;16(5):641–652. 24033570 10.1111/desc.12096 PMC3775298

[35] Gianaros PJ, Horenstein JA, Cohen S, et al Perigenual anterior cingulate morphology covaries with perceived social standing. Soc Cogn Affect Neurosci. 2007;2(3):161–173. 18418472 10.1093/scan/nsm013 PMC2312334

[36] Kishiyama MM, Boyce WT, Jimenez AM, Perry LM, Knight RT. Socioeconomic disparities affect prefrontal function in children. J Cogn Neurosci. 2009;21(6):1106–1115. 18752394 10.1162/jocn.2009.21101

[37] Huijsmans I, Ma I, Micheli L, Civai C, Stallen M, Sanfey AG. A scarcity mindset alters neural processing underlying consumer decision making. Proc Natl Acad Sci U S A. 2019;116(24):11699–11704. 31123150 10.1073/pnas.1818572116 PMC6575633

[38] Musslick S, Cohen JD. Rationalizing constraints on the capacity for cognitive control. Trends Cogn Sci. 2021;25(9):757–775. 34332856 10.1016/j.tics.2021.06.001

[39] Persson J, Welsh KM, Jonides J, Reuter-Lorenz PA. Cognitive fatigue of executive processes: Interaction between interference resolution tasks. Neuropsychologia. 2007;45(7):1571–1579. 17227678 10.1016/j.neuropsychologia.2006.12.007 PMC1876692

[40] Prickett KC, Augustine JM. Maternal education and investments in children’s health. J Marriage Fam. 2016;78(1):7–25. 26778853 10.1111/jomf.12253 PMC4712746

[41] Nampijja M, Kizindo R, Apule B, et al The role of the home environment in neurocognitive development of children living in extreme poverty and with frequent illnesses: A cross-sectional study. Wellcome Open Res. 2018;3:152. 30687794 10.12688/wellcomeopenres.14702.1 PMC6338129

[42] Fowler PJ, McGrath LM, Henry DB, et al Housing mobility and cognitive development: Change in verbal and nonverbal abilities. Child Abuse Negl. 2015;48:104–118. 26184055 10.1016/j.chiabu.2015.06.002 PMC4593721

[43] Baker E, Pham NTA, Daniel L, Bentley R. How does household residential instability influence child health outcomes? A quantile analysis. Int J Environ Res Public Health. 2019;16(21):4189. 31671903 10.3390/ijerph16214189 PMC6862481

[44] Holtzer C, Meaney FJ, Andrews J, et al Disparities in the diagnostic process of Duchenne and Becker muscular dystrophy. Genet Med. 2011;13(11):942–947. 21836521 10.1097/GIM.0b013e31822623f1

[45] Counterman KJ, Furlong P, Wang RT, Martin AS. Delays in diagnosis of Duchenne muscular dystrophy: An evaluation of genotypic and sociodemographic factors. Muscle Nerve. 2020;61(1):36–43. 31573675 10.1002/mus.26720

[46] Bushby K, Raybould S, O’Donnell S, Steele JG. Social deprivation in Duchenne muscular dystrophy: Population based study. BMJ. 2001;323(7320):1035–1036. 11691762 10.1136/bmj.323.7320.1035 PMC59456

