# Supplementary material for: Influence of community disadvantage on cognitive performance in son-mother dyads in Duchenne muscular dystrophy
Source: Brain Commun. 2026 Aug 20;8(4):fcag306. doi: 10.1093/braincomms/fcag306 (PMC13504700; doi:10.1093/braincomms/fcag306)
Supplement: Supplementary file 1 [file fcag306_supplementary_data.pdf]

Statistical codes

````
---
title: "Supplementary Material for Community Disadvantage and Cognitive Performance
  in Son-Mother Dyads in DMD"
author: "Karra et al."
output:
  html_document:
    toc: true
    toc_float: true
---

```{r setup, include=FALSE}
knitr::opts_chunk$set(echo = TRUE,
                      warning = FALSE,
                      message = FALSE)

```

# Supplementary Material for Community Disadvantage and Cognitive Performance in Son-
Mother Dyads in DMD

The provided script accompanies the manuscript by Karra et al. on the relationship
between community disadvantage and cognitive performance in son-mother dyads in
Duchenne Muscular Dystrophy (DMD).

## Set-Up Libraries and Fetch Data

```{r getData}

library(tidyverse)
library(readxl)
library(patchwork)

setwd("P:/DMD and Toolbox/Sept2024Data")

DMD_data <- read_excel("./DyadData-Final-square_updated.xlsx",
                       na=c("","na","n/a","na/", "NA","N/A","missing","incomplete
form"),
                       col_types = "text")
DMD_data <- DMD_data |>
  type_convert(cols(ChildID = col_character(),
                    MomID = col_character(),
                    .default = col_guess()))

DMD_data |>
  count(Instrument)

DMD_data <- DMD_data |>
  mutate(shortName = case_when(str_detect(Instrument, "Dimensional") ~ "DCCS",
                               str_detect(Instrument, "Flanker") ~ "Flanker",
                               str_detect(Instrument, "List Sor") ~ "LSWM",
                               str_detect(Instrument, "Oral Rea") ~ "ORR",
                               str_detect(Instrument, "Pattern Com") ~ "PCPS",
                               str_detect(Instrument, "Picture Seq") ~ "PSM",
                               str_detect(Instrument, "Picture Vocab") ~ "PVT",
                               str_detect(Instrument, "Crystallized") ~ "Crys",
                               str_detect(Instrument, "Fluid") ~ "Fluid",
````
````

````
                               str_detect(Instrument, "Total") ~ "Total",
                               str_detect(Instrument, "Early Child") ~ "ECComp",),
         DMDCoding = if_else(DMDCoding == "x", NA_character_, DMDCoding),
         Carrier2 = case_when(Carrier %in% c('yes','Yes') ~ "yes",
                              Carrier == "No" ~ "no",
                              TRUE ~ NA_character_)) |>
  type_convert(cols(ChildID = col_character(),
                    MomID = col_character(),
                    DMDCoding = col_number(),
                    .default = col_guess()))

```
````

Analyses

Description of Performance Overall for Boys and their Mothers

This code will summarize the performance for the boys and for their mothers (including non-biological mothers), with the mean/sd (as in the text of the manuscript) and the median and interquartile values, which correspond to the boxplots in Figure 1.

````
```{r overallPerformance}

overall_performance <- DMD_data |>
  filter(shortName != "ECComp") |>
  select(ChildID, MomID, shortName, Carrier2, ChildAgeSS, MomFCTscr) |>
  pivot_longer(cols = c(ChildAgeSS, MomFCTscr),
               names_to = "Measure", values_to = "Score") |>
  mutate(respondentID = if_else(Measure == "ChildAgeSS", ChildID, MomID)) |>
  select(-c(ChildID, MomID)) |>
  filter(!is.na(Score)) |>
  distinct() |> # keep only one set of scores for mothers of multiple children
  group_by(shortName, Measure) |>
  summarise(n=n(),
            meanScore = mean(Score),
            sdScore = sd(Score),
            medianScore = median(Score),
            q25 = quantile(Score, probs=.25),
            q75 = quantile(Score, probs=.75)) |>
  ungroup() |>
  arrange(Measure, shortName)

knitr::kable(overall_performance, digits=3, caption="Overall performance")

```
````

Comparison of Boys' Performance by DMD Coding

Wilcox tests were conducted by DMD status for each of the 7 measures and for the 3 composites. This is Table 2 in the manuscript.

````
```{r DMDCodingAnalysis}

analysis_coding <- DMD_data |>
  filter(!is.na(ChildAgeSS) & shortName != "ECComp") |>
  nest(.by=shortName) |>
````

```
  rowwise() |>
  mutate(modCarr = list(try(wilcox.test(ChildAgeSS ~ DMDCoding, conf.int=T, exact=F,
data=data))),
         g1count = length(data$ChildAgeSS[which(data$DMDCoding==1)]),
         g2count = length(data$ChildAgeSS[which(data$DMDCoding==2)]),
         g1median = median(data$ChildAgeSS[which(data$DMDCoding==1)]),
         g1iqr = IQR(data$ChildAgeSS[which(data$DMDCoding==1)]),
         g1min = min(data$ChildAgeSS[which(data$DMDCoding==1)]),
         g1max = max(data$ChildAgeSS[which(data$DMDCoding==1)]),
         g2median = median(data$ChildAgeSS[which(data$DMDCoding==2)]),
         g2iqr = IQR(data$ChildAgeSS[which(data$DMDCoding == 2)]),
         g2min = min(data$ChildAgeSS[which(data$DMDCoding==2)]),
         g2max = max(data$ChildAgeSS[which(data$DMDCoding==2)])) |>
  mutate(modCarr_out = list(broom::tidy(modCarr))) |>
  unnest(modCarr_out) |>
  select(shortName, starts_with("g1"), starts_with("g2"),
         statistic, p.value, estimate, conf.low, conf.high)

knitr::kable(analysis_coding, digits=3, caption="Wilcox tests by DMD coding;
g1=Proximal, g2=Distal.")
```

G1 = Proximal Mutation
G2 = Distal Mutation

Comparison of Mother's Performance by Carrier Status

These analyses are restricted to biological mothers with known carrier status. Wilcox tests were conducted by DMD status for each of the 7 measures and for the 3 composites. This is Table 3 in the manuscript.

```{r CarrierAnalysis}

analysis_carrier <- DMD_data |>
  filter(!is.na(MomFCTscr) & shortName != "ECComp" & !is.na(Carrier2)) |>
  select(MomID, shortName, Carrier2, MomFCTscr) |>
  distinct() |> # keep only one set of scores for mothers of multiple children
  nest(.by=shortName) |>
  rowwise() |>
  mutate(modCarr = list(try(wilcox.test(MomFCTscr ~ Carrier2, conf.int=T, exact=F,
data=data))),
         g1count = length(data$MomFCTscr[which(data$Carrier2=="yes")]),
         g2count = length(data$MomFCTscr[which(data$Carrier2=="no")]),
         g1median = median(data$MomFCTscr[which(data$Carrier2=="yes")]),
         g1iqr = IQR(data$MomFCTscr[which(data$Carrier2=="yes")]),
         g1min = min(data$MomFCTscr[which(data$Carrier2=="yes")]),
         g1max = max(data$MomFCTscr[which(data$Carrier2=="yes")]),
         g2median = median(data$MomFCTscr[which(data$Carrier2=="no")]),
         g2iqr = IQR(data$MomFCTscr[which(data$Carrier2 == "no")]),
         g2min = min(data$MomFCTscr[which(data$Carrier2=="no")]),
         g2max = max(data$MomFCTscr[which(data$Carrier2=="no")])) |>
  mutate(modCarr_out = list(broom::tidy(modCarr))) |>
  unnest(modCarr_out) |>
  select(shortName, starts_with("g1"), starts_with("g2"),
         statistic, p.value, estimate, conf.low, conf.high)
```

````
knitr::kable(analysis_carrier, digits=3, caption="Wilcox tests by Carrier status;
g1=Carrier, g2=Non-Carrier.")

```

G1 = Carrier
G2 = Non-Carrier

## Plot Generation

### Figure 2 - All Subjects - Profile Plot

```{r fig2}


figure2_panelA <- DMD_data |>
  mutate(Domain = case_when(shortName == "Total" ~ "Total",
                            shortName %in% c("Crys", "ORR","PVT") ~ "Crystalized",
                            shortName == "ECComp" ~ NA_character_,
                            TRUE ~ "Fluid"),
         shortName = factor(shortName,
levels=c("PVT","ORR","DCCS","Flanker","PCPS","PSM","LSWM",
                                                "Total","Crys","Fluid", "ECComp")))
|>
  filter(shortName %in% c("Total","Fluid","Crys")) |>
  ggplot(aes(x=shortName, y=ChildAgeSS, fill=Domain)) +
  scale_fill_manual(values=c("Total" = "#cc79a7", "Crystalized" = "#f0e442", "Fluid"
= "#d55e00")) +
  geom_boxplot(notch=F) + xlab("") + ylab("Standard Score") + ylim(50,150) +
  geom_hline(yintercept=100, linetype="solid", lwd=1.5)


figure2_panelB <- DMD_data |>
  mutate(Domain = case_when(shortName == "Total" ~ "Total",
                            shortName %in% c("Crys", "ORR","PVT") ~ "Crystalized",
                            shortName == "ECComp" ~ NA_character_,
                            TRUE ~ "Fluid"),
         shortName = factor(shortName,
levels=c("PVT","ORR","DCCS","Flanker","PCPS","PSM","LSWM",
                                                "Total","Fluid","Crys", "ECComp")))
|>
  filter(!shortName %in% c("Total","Fluid","Crys", "ECComp")) |>
  ggplot(aes(x=shortName, y=ChildAgeSS, fill=Domain)) +
  scale_fill_manual(values=c("Total" = "#cc79a7", "Crystalized" = "#f0e442", "Fluid"
= "#d55e00")) +
  geom_boxplot(notch=F) + xlab("") + ylab("") + ylim(50,150) +
  geom_hline(yintercept=100, linetype="solid", lwd=1.5) +
  theme(legend.position = "none")

figure2_panelC <- DMD_data |>
  mutate(Domain = case_when(shortName == "Total" ~ "Total",
                            shortName %in% c("Crys", "ORR","PVT") ~ "Crystalized",
                            shortName == "ECComp" ~ NA_character_,
                            TRUE ~ "Fluid"),
         shortName = factor(shortName,
levels=c("PVT","ORR","DCCS","Flanker","PCPS","PSM","LSWM",
                                                "Total","Crys","Fluid", "ECComp")),
         Carrier = as.factor(case_when(is.na(Carrier) ~ NA_character_,
````

````
                                        Carrier %in% c("Yes","yes") ~ "2_yes",
                                        Carrier == "No" ~ "1_no",
                                        TRUE ~ "0_unknown")) ) |>
  filter(shortName %in% c("Total","Fluid","Crys") &
           !is.na(Carrier)) |>
  ggplot(aes(x=shortName, y=MomFCTscr, fill=Domain)) +
  scale_fill_manual(values=c("Total" = "#cc79a7", "Crystalized" = "#f0e442", "Fluid"
= "#d55e00")) +
  geom_boxplot(notch=F) + xlab("") + ylab("Fully-Adjusted\nT-Score") + ylim(17,83) +
  geom_hline(yintercept=50, linetype="solid", lwd=1.5)

figure2_panelD <- DMD_data |>
  mutate(Domain = case_when(shortName == "Total" ~ "Total",
                            shortName %in% c("Crys", "ORR","PVT") ~ "Crystalized",
                            shortName == "ECComp" ~ NA_character_,
                            TRUE ~ "Fluid"),
         shortName = factor(shortName,
levels=c("PVT","ORR","DCCS","Flanker","PCPS","PSM","LSWM",
                                                  "Total","Fluid","Crys", "ECComp")),
         Carrier = as.factor(case_when(is.na(Carrier) ~ NA_character_,
                                        Carrier %in% c("Yes","yes") ~ "2_yes",
                                        Carrier == "No" ~ "1_no",
                                        TRUE ~ "0_unknown")) ) |>
  filter(!shortName %in% c("Total","Fluid","Crys", "ECComp") &
           !is.na(Carrier)) |>
  ggplot(aes(x=shortName, y=MomFCTscr, fill=Domain)) +
  scale_fill_manual(values=c("Total" = "#cc79a7", "Crystalized" = "#f0e442", "Fluid"
= "#d55e00")) +
  geom_boxplot(notch=F) + xlab("") + ylab("") + ylim(17,83) +
  geom_hline(yintercept=50, linetype="solid", lwd=1.5)  + theme(legend.position =
"none")


figure2 <- (figure2_panelA + figure2_panelB) / (figure2_panelC + figure2_panelD) +
  plot_annotation(tag_levels='A') + plot_layout(guides = "collect")

figure2


```
````

Note: for the publication, this figure adds ` & theme(plot.margin = margin(5.5, 5.5, 5.5, 5.5))` after the `plot_layout()` command, but this syntax does not work in the Rmarkdown environment.

Figure 3 - All Subjects - Profile Plot by DMD Coding

````
```{r fig3}


figure3_panelA <-  DMD_data |>
  mutate(Domain = case_when(shortName == "Total" ~ "Total",
                            shortName %in% c("Crys", "ORR","PVT") ~ "Crystalized",
                            shortName == "ECComp" ~ NA_character_,
                            TRUE ~ "Fluid"),
         shortName = factor(shortName,
levels=c("PVT","ORR","DCCS","Flanker","PCPS","PSM","LSWM",
                                                  "Total","Crys","Fluid", "ECComp")),
````

```
         DMDCoding = as.factor(DMDCoding)) |>
  filter(shortName %in% c("Total","Fluid","Crys") &
           !is.na(DMDCoding)) |>
  ggplot(aes(x=shortName, y=ChildAgeSS, fill=Domain, color=DMDCoding)) +
  scale_fill_manual(values=c("Total" = "#cc79a7", "Crystalized" = "#f0e442", "Fluid"
= "#d55e00")) +
  scale_color_manual(values=c("1" = "#009e73", "2" = "#0072b2")) +
  geom_boxplot(notch=F, linewidth=1) + xlab("") + ylab("Standard Score") +
ylim(50,150) +
  geom_hline(yintercept=100, linetype="solid", lwd=1.5)

figure3_panelB <- DMD_data |>
  mutate(Domain = case_when(shortName == "Total" ~ "Total",
                            shortName %in% c("Crys", "ORR","PVT") ~ "Crystalized",
                            shortName == "ECComp" ~ NA_character_,
                            TRUE ~ "Fluid"),
         shortName = factor(shortName,
levels=c("PVT","ORR","DCCS","Flanker","PCPS","PSM","LSWM",
                                                 "Total","Fluid","Crys", "ECComp")),
         DMDCoding = as.factor(DMDCoding)) |>
  filter(!shortName %in% c("Total","Fluid","Crys", "ECComp") &
           !is.na(DMDCoding)) |>
  ggplot(aes(x=shortName, y=ChildAgeSS, fill=Domain, color=DMDCoding)) +
  scale_fill_manual(values=c("Total" = "#cc79a7", "Crystalized" = "#f0e442", "Fluid"
= "#d55e00")) +
  scale_color_manual(values=c("1" = "#009e73", "2" = "#0072b2")) +
  geom_boxplot(notch=F, linewidth=1) + xlab("") + ylab("") + ylim(50,150) +
  geom_hline(yintercept=100, linetype="solid", lwd=1.5) + theme(legend.position =
'none')

figure3 <- figure3_panelA + figure3_panelB +
  plot_annotation(tag_levels='A') + plot_layout(guides = "collect")


figure3

```

Note: for the publication, this figure adds ` & theme(plot.margin = margin(5.5, 5.5, 5.5, 5.5))` after the `plot_layout()` command, but this syntax does not work in the Rmarkdown environment.

Figure 4 - All Subjects - Profile Plot by Carrier Status

```{r fig4}

figure4_panelA <- DMD_data |>
  mutate(Domain = case_when(shortName == "Total" ~ "Total",
                            shortName %in% c("Crys", "ORR","PVT") ~ "Crystalized",
                            shortName == "ECComp" ~ NA_character_,
                            TRUE ~ "Fluid"),
         shortName = factor(shortName,
levels=c("PVT","ORR","DCCS","Flanker","PCPS","PSM","LSWM",
                                                 "Total","Crys","Fluid", "ECComp")),
         Carrier = as.factor(case_when(is.na(Carrier) ~ NA_character_,
                                       Carrier %in% c("Yes","yes") ~ "2_yes",
                                       Carrier == "No" ~ "1_no",
                                       TRUE ~ NA_character_)) ) |>
```

```
  filter(shortName %in% c("Total","Fluid","Crys") &
           !is.na(Carrier)) |>
  ggplot(aes(x=shortName, y=MomFCTscr, fill=Domain, color=Carrier)) +
  scale_fill_manual(values=c("Total" = "#cc79a7", "Crystalized" = "#f0e442", "Fluid"
= "#d55e00")) +
  scale_color_manual(values=c("1_no" = "#998ec3", "2_yes" = "#542788")) +
  geom_boxplot(notch=F, linewidth=1) + xlab("") + ylab("Fully-Adjusted\nT-Score") +
ylim(17,83) +
  geom_hline(yintercept=50, linetype="solid", lwd=1.5)

figure4_panelB <- DMD_data |>
  mutate(Domain = case_when(shortName == "Total" ~ "Total",
                            shortName %in% c("Crys", "ORR","PVT") ~ "Crystalized",
                            shortName == "ECComp" ~ NA_character_,
                            TRUE ~ "Fluid"),
         shortName = factor(shortName,
levels=c("PVT","ORR","DCCS","Flanker","PCPS","PSM","LSWM",
                                               "Total","Fluid","Crys", "ECComp")),
         Carrier = as.factor(case_when(is.na(Carrier) ~ NA_character_,
                                       Carrier %in% c("Yes","yes") ~ "2_yes",
                                       Carrier == "No" ~ "1_no",
                                       TRUE ~ NA_character_)) ) |>
  filter(!shortName %in% c("Total","Fluid","Crys", "ECComp") &
           !is.na(Carrier)) |>
  select(MomID, Domain, shortName, Carrier, MomFCTscr) |>
  distinct() |> # keep only one set of scores for mothers of multiple children
  ggplot(aes(x=shortName, y=MomFCTscr, fill=Domain, color=Carrier)) +
  scale_fill_manual(values=c("Total" = "#cc79a7", "Crystalized" = "#f0e442", "Fluid"
= "#d55e00")) +
  scale_color_manual(values=c("1_no" = "#998ec3", "2_yes" = "#542788")) +
  geom_boxplot(notch=F, linewidth=1) + xlab("") + ylab("") + ylim(17,83) +
  geom_hline(yintercept=50, linetype="solid", lwd=1.5) + theme(legend.position =
'none')

figure4 <- figure4_panelA + figure4_panelB +
  plot_annotation(tag_levels='A') + plot_layout(guides = "collect")

figure4

```

Note: for the publication, this figure adds ` & theme(plot.margin = margin(5.5, 5.5, 5.5, 5.5))` after the `plot_layout()` command, but this syntax does not work in the Rmarkdown environment.

Figure 5 - Relationship Between Social Vulnerability Index (SVI) and Cognitive Performance

Statistically-significant relationships were found between SVI and scores focused on Flanker for the boys, and DCCS for their mothers.

Note, unlike the earlier figures, these panels were not generated using the `patchwork` package, but combined outside of the statistical code.

```{r fig5a}

fig5a <- DMD_data |>
  filter(shortName == "Flanker" & !is.na(ChildAgeSS)) |>
```

~~~
  ggplot(aes(x=SVI_Score, y=ChildAgeSS)) + geom_point() + geom_smooth(method='lm') +
  scale_x_continuous(name="Social Vulnerability Index", breaks=seq(0,1.25,.25)) +
  ylab("Age-Adjusted Standard Score")
tmp <- DMD_data |>
  filter(shortName == "Flanker" & !is.na(ChildAgeSS)) |>
  select(SVI_Score, ChildAgeSS) |>
  cor(use='pairwise',method='pearson')

fig5a <- fig5a + annotate("text", x=0.675, y=105, label=paste0("r = ",
round(tmp[2,1],2)), size=5)
fig5a

```

```{r fig5b}

fig5b <- DMD_data |>
  filter(shortName == "DCCS" & between(MomFCTscr, 19, 81)) |>
  ggplot(aes(x=SVI_Score, y=MomFCTscr)) + geom_point() + geom_smooth(method='lm') +
  xlab("Social Vulnerability Index") + ylab("Fully-Adjusted T-Scores")

tmp <- DMD_data |>
  filter(shortName == "DCCS" & between(MomFCTscr, 19, 81)) |>
  select(SVI_Score, MomFCTscr) |>
  cor(use='pairwise',method='pearson')

fig5b <- fig5b + annotate("text", x=0.675, y=70, label= paste0("r = ",
round(tmp[2,1],2)), size=5)
fig5b

```
~~~
